# Tumor treating fields for newly diagnosed high‐grade glioma based on the criteria of 2021 WHO CNS5: A retrospective analysis of Chinese patients in a single center

**DOI:** 10.1002/cam4.7350

**Published:** 2024-06-10

**Authors:** Li Zhang, Yanming Ren, Youheng Peng, Yong Luo, Yanhui Liu, Xiang Wang, Yuan Yang, Lei Liu, Ping Ai, Xiaoyan Yang, Yanchu Li, Qing Mao, Feng Wang

**Affiliations:** ^1^ Head and Neck Oncology Ward, Cancer Center, West China Hospital Sichuan University Chengdu China; ^2^ Department of Neurosurgery, West China Hospital Sichuan University Chengdu China

**Keywords:** astrocytoma, glioblastoma, high‐grade glioma, tumor treating fields, WHO CNS4 2016, WHO CNS5 2021

## Abstract

**Background and Objective:**

High‐grade glioma (HGG) is known to be characterized by a high degree of malignancy and a worse prognosis. The classical treatment is safe resection supplemented by radiotherapy and chemotherapy. Tumor treating fields (TTFields), an emerging physiotherapeutic modality that targets malignant solid tumors using medium‐frequency, low‐intensity, alternating electric fields to interfere with cell division, have been used for the treatment of new diagnosis of glioblastoma, however, their administration in HGG requires further clinical evidence. The efficacy and safety of TTFields in Chinese patients with HGG were retrospectively evaluated by us in a single center.

**Methods:**

We enrolled and analyzed 52 patients with newly diagnosed HGG undergoing surgery and standard chemoradiotherapy regimens from December 2019 to June 2022, and followed them until June 2023. Based on whether they used TTFields, they were divided into a TTFields group and a non‐TTFields group. Progression‐free survival (PFS) and overall survival (OS) were compared between the two groups.

**Results:**

There were 26 cases in the TTFields group and 26 cases in the non‐TTFields group. In the TTFields group, the median PFS was 14.2 months (95% CI: 9.50–18.90), the median OS was 19.7 months (95% CI: 14.95–24.25) , the median interval from surgery to the start of treatment with TTFields was 2.47 months (95% CI: 1.47–4.13), and the median duration of treatment with TTFields was 10.6 months (95% CI: 9.57–11.63). 15 (57.69%) patients experienced an adverse event and no serious adverse event was reported. In the non‐TTFields group, the median PFS was 9.57 months (95% CI: 6.23–12.91) and the median OS was 16.07 months (95% CI: 12.90–19.24). There was a statistically significant difference in PFS (*p* = 0.005) and OS (*p* = 0.007) between the two groups.

**Conclusions:**

In this retrospective analysis, TTFields were observed to improve newly diagnosed HGG patients' median PFS and OS. Compliance was much higher than reported in clinical trials and safety remained good.

## 1 INTRODUCTION

Glioma is the most common type of intracranial tumor, characterized by invasive growth patterns, often involving surrounding tissues.^1^ As the most commonly diagnosed primary intracranial malignancy in adults, glioma has three prominent characteristics: high incidence, high disability rate, and high recurrence rate.^2^ According to the Chinese Multicentre Cross‐Sectional Study of Brain Tumors, the age‐standardized prevalence of primary brain tumors in the general population is estimated to be 22.52 million, with gliomas accounting for 31.1% of those aged 20–59 years. Among gliomas, diffuse and anaplastic astrocytomas account for approximately 25.2% of cases. Oligodendrocytoma and oligodendroglioma account for about 18% of gliomas, while glioblastoma (GBM) accounts for about 30% of central nervous system (CNS) gliomas.^3^ High‐grade glioma (HGG), which includes World Health Organization (WHO) grade 3/4 gliomas, is the most common form of primary malignant tumor in adults and is also one of the most lethal solid tumors in adults. HGG can occur in all age groups, including children, and its incidence increases with age.^2^

The management of HGG has evolved significantly over the past decade. Currently, the primary approach involves the widest possible surgical resection to ensure safety, followed by adjuvant radiotherapy and chemotherapy. For newly diagnosed GBM, the standard of care is radiotherapy after surgical resection or biopsy, combined with temozolomide (TMZ) chemotherapy, followed by TMZ maintenance.^4^ However, despite extensive research, randomized clinical trials investigating dose‐dense TMZ, bevacizumab, and everolimus have failed to demonstrate significant improvements in survival compared with the standard of care. As a result, median progression‐free survival (PFS) and median overall survival (OS) for newly diagnosed GBM remain modest at approximately 6.2–7.5 months and 14.6–16.7 months, respectively.^5^, ^6^, ^7^, ^8^ In addition, the lack of robust evidence leaves uncertainty regarding the potential benefits of reoperation or radiotherapy for patients with recurrent HGG.^9^ Therefore, there is an urgent and compelling need to develop new, safe, and effective treatments that can extend PFS and OS while preserving patients' limited quality of life.

Tumor treating field(TTFields) is an emerging physical therapy for cancer. Decades ago, scientists found that external electric fields with different frequencies can have different significant impacts on cell biology.^10^, ^11^ Initially, it was thought that intermediate‐frequency alternating current fields (KHz to MHz range) would not exert meaningful biological effects, but Eilon D. Kirson et al. found in a preclinical study carried out in 2004 that 100–500 KHz inhibited the proliferation of malignant glioma cell lines and the growth of in vivo tumor models in mice in a frequency, intensity, and time‐dependent.^12^, ^13^ These findings led to the development of TTFields to treat cancer.^14^ TTFields work by emitting a low‐intensity, intermediate‐frequency (200 kHz) alternating electric field, which is applied through an array of insulated transducers applied to the scalp. The electric field penetrates the brain and inhibits the growth and proliferation of GBM by interfering with late‐stage tumor cell mitosis.^12^, ^13^, ^14^ Interference with mitosis is believed to be the first identified mechanism of anticancer action of TTFields. TTFields interfere with mitosis in tumor cells by disrupting microtubulin polymerization, septal localization, and cytokinesis, preventing mitotic spindle assembly, and adjusting cellular membrane potential to increase the influx of intracellular Ca^2+^ to promote microtubulin depolymerization, which causes cell cycle arrest, and ultimately leads to tumor cell death/apoptosis.^15^, ^16^, ^17^, ^18^, ^19^, ^20^, ^21^, ^22^ The anti‐tumor mechanisms of action of TTFields are also associated with influencing tumor cell migration, increasing the permeability of tumor cell membranes and the blood‐brain barrier, inducing autophagy in tumor cells, increasing DNA damage, and inducing antitumor immunity.^23^, ^24^, ^25^, ^26^, ^27^, ^28^, ^29^, ^30^, ^31^ TTFields have been approved by the FDA for recurrent and newly diagnosed GBM based on the results of clinical studies in EF‐11 and EF‐14.^14^, ^32^ In addition, TTFields have been approved for the treatment of mesothelioma and are currently being studied in many other tumor types including non‐small cell lung cancer, pancreatic cancer, ovarian cancer and so on.^33^, ^34^, ^35^, ^36^ Notably, patients can seamlessly integrate TTFields therapy into their daily routines and demonstrate high treatment compliance.^14^

The fifth edition of the WHO Classification of Tumors of the CNS, published in 2021, represents the sixth iteration of the globally accepted standard for brain and spinal cord tumor classification. This latest edition introduces new approaches to CNS tumor nomenclature and grading, emphasizing the importance of integrated diagnoses and layered reporting.^37^ In particular, the revised definition of pathological diagnosis in the 2021 edition of the WHO CNS5 has potential implications for subsequent standard treatment regimens, such as the Stupp protocol. TTFields are widely used abroad, however, the administration of TTFields in HGG in China requires further clinical evidence. This study aims to retrospectively evaluate and analyze the safety, efficacy, and impact on the quality of life of TTFields for Chinese patients with HGG based on the criteria outlined in WHO CNS5 2021 after it has been marketed in China, to facilitate the related scientists to have a comprehensive and systematic understanding of the clinical practice of TTFields therapy in China.

## 2 METHODS

### 2.1 Study design and setting

The study was approved by the Ethics Committee on Biomedical Research, West China Hospital of Sichuan University, and a waiver of informed consent was obtained. We conducted a retrospective analysis of 52 patients diagnosed with HGG and treated at the West China Hospital, Sichuan University between December 2019 and June 2022 (Tables S1 and S2). They were categorized into groups with and without TTFields based on the use of TTFields or not. Relevant demographic and clinical data were collected and extracted from the institutional hospital information system.

All patients underwent routine follow‐up examinations for 1–2 months, with subsequent follow‐up examinations scheduled at least every 3 months until June 2023. Participants fulfilled the following inclusion criteria: (1) enrolled in a big data platform for intelligent management of the whole course of brain glioma patients; (2) histologically confirmed as HGG according to WHO grading standards; (3) tumor was supratentorial; (4) received TTFields treatment; (5) age ≥18 years; (6) if baseline data is complete, there should be at least 1 month of follow‐up data. Exclusion criteria were as follows: (1) pregnant women, lactating patients, and patients planning to become pregnant; (2) patients allergic to conductive hydrogel; (3) patients with large skull defects; (4) TTFields used for less than 4 weeks.

Baseline characteristics included: (1) sex; (2) age; (3)baseline Karnofsky performance status (KPS) score; (4) pathological type and WHO grade (according to the 2016 and 2021) WHO classification criteria for CNS tumors, respectively; (5) tumor location; (6) O6‐methylguanine DNA methyltransferase (MGMT) methylation status (methylation, non‐methylation, unknown); (7) other gene marker indicators: a. EGFR amplification, overexpression or rearrangement; b. IDH1 mutation; c. b. Chromosome 1p/19q deletion status; d. TERT promoter mutation; (8) TTFields usage; (9) PFS; (10) OS; (11) whether chemoradiation treatment with concurrent TTFields; (12) compliance with TTFields treatment; (13) duration of TTFields treatment.

TTFields requires professional healthcare workers or device administrators to instruct the patient on how to use the device, replace the electric field patches, and recharge and replace the batteries. To use the NovaTTFields‐200A device (Novocure, Israel), the patient's scalp is shaved, and four electric field patches are placed on the scalp to generate low‐intensity (2 V/cm), intermediate‐frequency (200 kHz), and alternating electric fields in the tumor area. Patients were encouraged to place the patches for more than 18 hours (h) per day. The electric field patches were replaced twice a week. The PFS of patients with newly diagnosed HGG was defined as the time from surgery to tumor progression or surgical death of the patient. The OS was defined as the time from surgery to the patient's death or cutoff follow‐up date. The incidence of skin adverse events in our patients with newly diagnosed HGG treated with TTFields was evaluated using the Common Terminology Criteria for Adverse Events (CTCAE) version 5.0 and the TTFields skin reaction‐related scoring system (Tables 1 and 2).^37^, ^38^ Subjects' compliance during follow‐up is reflected by the average daily use time of a single course of treatment, and the device support specialist will download the usage file stored in the TTFields device at least once every 3 months, which records the cumulative time that patients normally receive treatment each day, thus assessing each patient's compliance. Compliance, which corresponds to the average daily use time within a month from low to high, is 0–30% (<7 h), 30%–50% (7.2–12 h), 50%–60% (12–14.4 h), 60%–75% (14.4–18 h), 75%–90% (18–21.6 h), 90%–100% (>24 h). Actual usage data can be obtained from the treatment device.

**TABLE 1:** CTCAEV5.0.

Grade	Description
1	Mild; asymptomatic or mild symptoms; clinical or diagnostic observations only; intervention not indicated
2	Moderate; minimal, local, or noninvasive intervention indicated; limiting age‐appropriate instrumental ADLs^a^
3	Severe or medically significant but not immediately life‐threatening; hospitalization or prolongation of hospitalization indicated; disabling; limiting self‐care ADLs^b^
4	Life‐threatening consequences; urgent intervention indicated
5	Death related to AE

^a^ Instrumental ADLs refer to preparing meals, shopping for groceries or clothes, using the telephone, managing money, etc.

^b^ Self‐care ADLs refer to bathing, dressing, undressing, feeding self, using the toilet, taking medications, and not being bedridden.

**TABLE 2:** TTFields skin reaction‐related scoring system.^38^

Grade	Description
1	No symptoms or mild symptoms
2	Moderate symptoms; Need local and systemic treatment (such as antibiotics and corticosteroids); The equipment needs to be suspended; The electrode patch position needs to be adjusted temporarily to avoid the affected skin area; The affected area needs dressing isolation
3	Serious or significant clinical symptoms; Need local and systemic treatment (such as antibiotics and corticosteroids), but do not immediately endanger life; Prompt for surgical intervention; Need to be hospitalized or extend the existing hospitalization; Equipment use interruption
4	Life‐threatening effects: there is a prompt for emergency intervention and a prompt for disabling the device

### 2.2 Statistical analysis

Enumeration data were described by frequency (%), and analyzed by chi‐squared or Fisher's exact test. Measurement data that conformed to normal distribution were described by mean followed by standard deviation, and were analyzed by Student's *t*‐test and Pearson's correlation analysis; measurement data that did not conform to normal distribution were described by median, minimum, and maximum, and analyzed by Mann–Whitney *U*‐test and the Spearman's rank coefficient of correlation analysis. The median PFS and OS were analyzed using the Kaplan–Meier survival curves, and the difference in survival curves was tested by the Log‐Rank method. *p* < 0.05 was considered statistically significant.

Data processing was performed using GraphPad Prism 8 (GraphPad Software, La Jolla, CA, USA), SPSS 26.0 (IBM Corporation, Armonk, New York, USA) software, and R software version 4.2.2 (R Foundation for Statistical Computing; http://www. R‐project.org, 2017).

## 3 RESULTS

A total of 52 patients were included in the analysis, and all of these 52 patients underwent surgery with maximal safe resection and a standard chemoradiotherapy regimen. In the TTFields group, there were 26 patients, including 17 males and 9 females, with a mean age of 52.15 ± 10.89 years, and the median preoperative KPS score was 100. In the non‐TTFields group, there were 26 patients, including 15 males and 11 females, with a mean age of 56.67 ± 13.85 years, and the median preoperative KPS score was 100. The differences in baseline characteristics, gender, age, KPS score, tumor location, MGMT promoter, IDH mutation status, EGFR status, 1p/19q loss status, and TERT promoter status between the two groups were not statistically significant (all *p* > 0.05). Table 3 provides detailed information on the baseline and molecular pathological characteristics of the patient cohort.

**TABLE 3:** Clinical characteristics of HGG patients.

Characteristics	Number (%) of patients		
	TTFields group (*n* = 26)	Non‐TTFields group (*n* = 26)	*p*‐values
Sex			
Male	17 (65.38%)	15 (57.69%)	0.569
Female	9 (34.62%)	11 (42.31%)	
Age at diagnosis	52.15 ± 10.89	56.67 ± 13.85	0.188
Median preoperative KPS score	100 (50–100)	95 (60–100)	0.944
Tumor location			
Frontal lobe	13 (50%)	12 (46.15%)	0.980
Temporal lobe	3 (11.54%)	5 (19.23%)	
Parietal lobe	3 (11.54%)	2 (7.69%)	
Occipital lobe	2 (7.69%)	1 (3.85%)	
Basal ganglia	2 (7.69%)	2 (7.69%)	
Thalamus	2 (7.69%)	3 (11.54%)	
Others	1 (3.85%)	1 (3.85%)	
Pathological type (2016 WHO CNS4)			
Glioblastoma	20 (76.92%)	19 (73.08%)	0.536
Anaplastic astrocytoma	2 (7.69%)	5 (19.23%)	
Anaplastic oligodendroglioma	NA	NA	
Diffuse midline glioma	3 (11.54%)	5 (19.23%)	
Diffuse astrocyte glioma	1 (3.85%)	NA	
WHO classification (2016 WHO CNS4)			
WHO 3	2 (7.69%)	4 (15.38%)	0.664
WHO 4	24 (92.31%)	22 (84.62%)	
Pathological type (2021WHO CNS5)			
Glioblastoma	11 (42.31%)	17 (65.38%)	0.686
Astrocytoma	9 (34.62%)	7 (26.9211%)	
Oligodendroglioma	3 (11.54%)	NA	
Diffuse midline glioma	3 (11.54%)	2 (7.69%)	
WHO classification (2021 WHO CNS5)			
NEC/NOC	3 (11.54%)	NA	0.319
WHO 3	2 (7.69%)	2 (7.41%)	
WHO 4	21 (80.77%)	24 (92.59%)	
MGMT promoter methylation			
Methylated	13 (50.00%)	12 (46.15%)	1.000
Unmethylated	12 (46.15%)	12 (46.15%)	
Unknown	1 (3.85%)	2 (7.69%)	
EGFR amplification, overexpression or rearrangement			
Yes	11 (42.31%)	7 (26.92%)	0.280
No	3 (11.54%)	1 (3.85%)	
Unknown	12 (46.15%)	18 (69.23%)	
IDH1/2 mutation state			
Wild‐type	16 (29.26%)	18 (69.23%)	0.778
Mutant	4 (14.81%)	2 (7.69%)	
Unknown	7 (25.93%)	6 (23.08%)	
1p/19q deletions			
Co‐deleted	NA	NA	1.000
Non‐co‐deleted	5 (19.23%)	4 (15.38%)	
Unknown	21 (80.77%)	22 (84.62%)	
TERT promoter mutation			
Yes	18 (66.23%)	17 (65.38%)	0.266
No	8 (30.77%)	6 (23.08%)	
Unknown	NA	3 (11.54%)	
AE occurs, *n* (%)			
Yes	15 (55.6%)		
No	11 (40.7%)		
Unknown	1 (3.7%)		
Median compliance	91.50% (60%–100%)		
Median TTFields treatment time, months	10.60 (9.57–11.63)		

**FIGURE 1:**
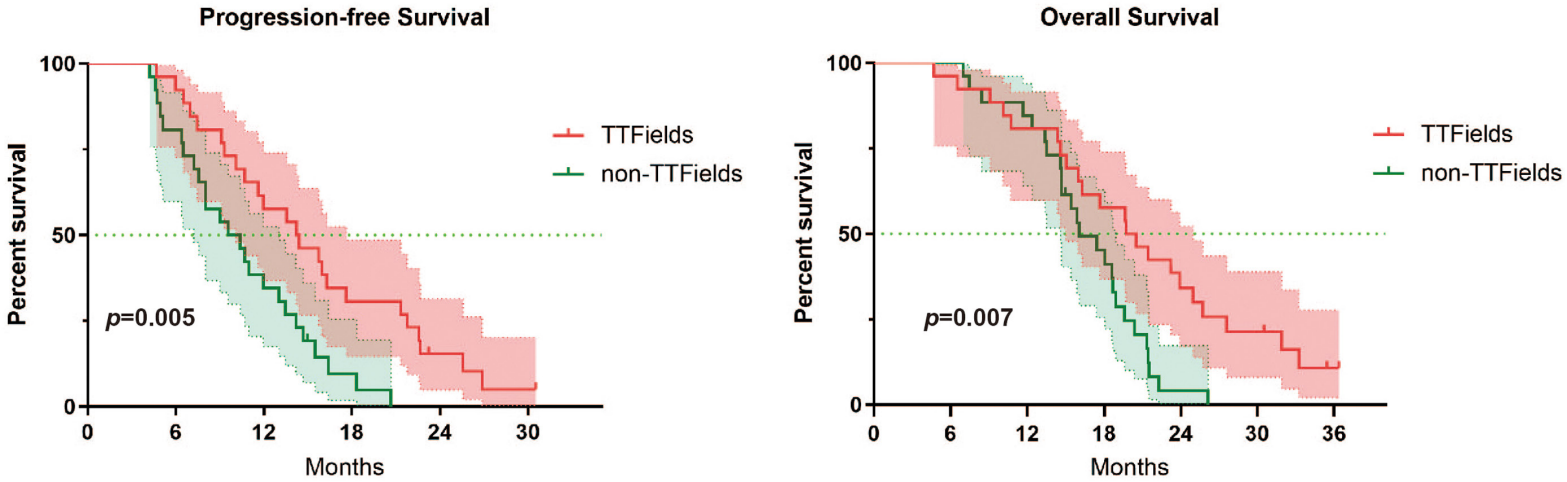
Kaplan–Meier curves of PFS and OS for newly diagnosed high‐grade glioma patients in two groups.

**FIGURE 2:**
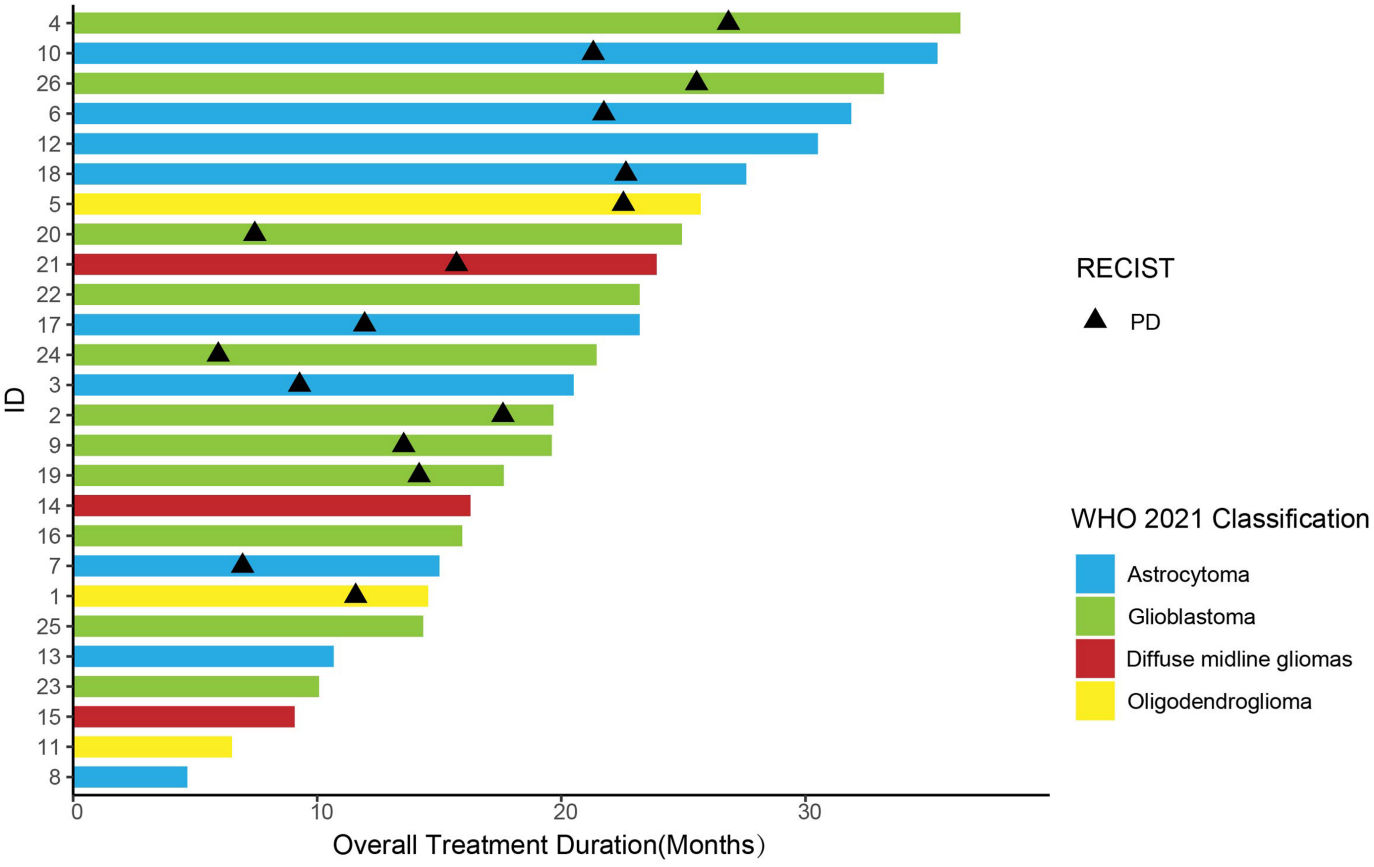
The survival of 26 patients with disease progression or death or by follow‐up time starting from the date of surgery.

In the TTFields group, the median interval between surgery and initiation of TTFields treatment was 2.47 months (95% CI: 1.47–4.13); the median duration of treatment with TTFields was 10.6 months (95% CI: 9.57–11.63); the median compliance of TFields treatment was 91.50%; the median PFS was 14.2 months (95% CI: 9.50–18.90), and the median OS was 19.7 months (95% CI: 14.95–24.25). The non‐TTFields group's median PFS was 9.57 months (95% CI: 6.23–12.91), and the median OS was 16.07 months (95% CI: 12.90–19.24). Figure 1 shows the Kaplan–Meier curves for PFS and OS. Figure 2 shows the survival of the 26 patients based on disease progression, death, or follow‐up time from the date of surgery. In addition, patients are differentiated according to the different pathological definitions derived from the WHO 2021 CNS5 classification.

15 out of 26 patients (57.69%) experienced AE in the TTFields group. However, all AE were limited to skin reactions, specifically rash or erythema. Based on the CTCAE version 5.0 and the TTFields skin reaction scoring system, all scalp adverse reactions, except for one case, were classified as grade one. At the time of the last follow‐up, 5 patients showed improvement in scalp adverse reactions, with an average treatment duration of 67.8 days. No cases of SAE were reported during the study. Furthermore, up until the data cut‐off point, 21 deaths were recorded, none of which were determined to be related to the use of the TTFields device.

Of the 26 patients in the TTFields group included in the study, 20 were diagnosed with GBM, which is grade 4 according to the 2016 WHO CNS4 criteria. On the other hand, 11 patients were diagnosed with GBM, and 9 patients were diagnosed with astrocytoma according to the 2021 WHO CNS5 criteria.

Correlation analysis showed that the two groups' OS was not significantly correlated with age, gender, KPS score, MGMT status, IDH status, EGFR status, 1p/19q deletions status, and TERT promoter status(all *p* > 0.5). However, the OS of the TTFields group showed a significant positive correlation with the duration of TTFields treatment (*ρ* = 0.555, *p* = 0.003). Similarly, the PFS of the TTFields group was only associated with the duration of TTFields treatment (*ρ* = 0.414, *p* = 0.036). A negative correlation was also observed between patient compliance and the occurrence of AEs (*ρ* = −0.323, *p* = 0.107).

## 4 DISCUSSION

Current treatments for HGG are still limited to surgery combined with concurrent chemoradiotherapy and chemo‐maintenance, however, TTFields, a wearable physical therapy, has no significant systemic side effects, can remarkably prolong survival and improve quality of life for patients after completion of standard radio‐chemotherapy, which provides a therapeutic option for patients.^39^ Due to its favorable tolerability, TTFields therapy can be combined with maintenance chemotherapy or targeted therapy in patients with newly diagnosed brain tumors following surgical resection and radiotherapy.^40^

As a non‐invasive and anti‐mitotic treatment modality, TTFields received FDA approval in 2011 for recurrent GBM and in 2015 for newly diagnosed GBM. It has since been used in clinical practice in Europe, Japan, and Hong Kong, China. This retrospective study, conducted in a real‐world clinical setting, aimed to evaluate the safety and efficacy of TTFields therapy in patients with HGG. The baseline characteristics of the study population indicate that it is representative of the real‐world GBM population in terms of male/female ratio and mean age.^41^

AEs associated with TTFields therapy primarily manifest as dermatologic toxicities, primarily localized reactions beneath the device array. These reactions commonly include contact dermatitis, hyperhidrosis, dryness, and pruritus and typically occur within 2–6 weeks of TTFields use.^42^ The majority of skin‐related AEs are classified as mild to moderate (grade 1/2), while a small percentage of patients (only 2% of EF‐14 participants) may experience severe skin damage (grade ≥3 AEs).^43^ In our study, all reported AEs presented as mild rash or erythema, with no occurrence of serious skin reactions such as erosions or ulcers. This result may be attributed to our comprehensive care process. Before starting treatment with TTFields, subjects, and their caregivers receive specialized training from a qualified physician or device support specialist to ensure proper device operation and provide guidance on daily care practices. During the first month of treatment, a minimum of 2–3 close follow‐up visits are performed to reinforce correct device operation and care procedures and to monitor scalp reactions. Subsequent follow‐up visits occur at least once every 3 months. Throughout the follow‐up period, the clinician evaluates the subject's scalp condition in person or based on at least four scalp photographs provided by the subject. In addition, subjects can report scalp side effects through the system at any time during the non‐follow‐up periods, and clinicians respond within 24 h to provide treatment recommendations and maintain appropriate records.

A meta‐analysis of skin toxicity associated with TTFields found that 48% of TTFields users experienced adverse skin reactions,^44^ which is consistent with the results of our study. Skin reactions associated with TTFields therapy can usually be effectively managed with simple precautions. Common interventions include the use of topical corticosteroids for irritant or contact dermatitis and topical antibiotics for skin ulcers, administered during array changes.^38^, ^42^ It is also important for patients to ensure that their scalp is completely dry before applying new arrays. Implementing preventive measures, such as using an electric razor instead of razor blades to minimize the risk of cuts and folliculitis, and regularly repositioning the array, can further reduce the likelihood of skin rupture.^45^, ^46^ Importantly, similar to the majority of previous studies, no treatment‐related SAEs were reported in our study.^11^, ^44^, ^47^

The efficacy of TTFields therapy is primarily dependent on patient compliance, which is critical to achieving optimal results. It typically takes a minimum of 4 weeks to observe substantial tumor growth reversal with TTFields treatment.^48^ Therefore, the prevention and management of TTFields‐related adverse events, particularly skin toxicity, play an important role in maintaining the quality of life of patients with GBM, ensuring continued use of TTFields, and maximizing clinical benefit.^38^ This correlation between compliance and skin reactions was further supported by the Pearson correlation coefficient analysis performed in our study. Furthermore, our study demonstrated a median compliance rate of 91.50% in the TTFields patient cohort, which may be attributed to the mild scalp side effects experienced by our patients. Previous studies have investigated the impact of compliance on the prognosis of patients with recurrent GBM and newly diagnosed GBM and have shown that daily compliance of ≥75% is associated with significantly increased median OS, PFS, and 1‐year survival, with improved prognosis correlating with higher compliance rates^13^, ^49^ In our study, we observed a median PFS of 14.20 months, a median OS of 19.70 months, all of which were significantly higher than patients receiving standard treatment alone in our study and those reported in other studies.^6^, ^7^ In addition, our study demonstrated improved PFS compared to other studies evaluating the combination of TTFields and TMZ for the treatment of GBM.^14^, ^50^ This improvement may be due to the higher compliance rates in our patients as well as the longer duration of treatment received.

Overall, our findings underscore the importance of patient compliance in achieving favorable outcomes with TTFields therapy. High compliance rates in our study were associated with improved survival outcomes, highlighting the potential benefits of sustained and rigorous adherence to TTFields treatment protocols.

## 5 LIMITATION

Unfortunately, despite the ongoing development of TTFields technology and the gradual reduction in its cost, it remains an expensive treatment, exceeding the price of conventional GBM treatment. As a result, our study was limited by a relatively small patient cohort, making it difficult to perform a comprehensive subgroup analysis to investigate factors influencing the efficacy of TTFields. To address this limitation, future studies will include a larger sample size to allow a more robust investigation of these factors.

Furthermore, our research was unable to objectively assess the quality of life of our patients due to the lack of appropriate assessment tools. As a result, the assessment of QoL in our study was subjective and relied on self‐report. To overcome this limitation, there is a need to develop user‐friendly and objective tools that can accurately assess the quality of life of patients undergoing TTFields therapy. Future research should focus on the identification and implementation of such tools to provide a more comprehensive assessment of patient outcomes and treatment efficacy.

Overall, while our study provides valuable insights into the efficacy and challenges of TTFields therapy, limitations in sample size and quality of life assessment highlight areas for further research and improvement.

## 6 CONCLUSION

GBM, the most common primary intracranial malignancy in adults, remains one of the deadliest solid tumors. The use of TTFields has demonstrated the ability to inhibit the proliferation of malignant tumor cell lines, resulting in improved survival and quality of life for GBM patients. Currently, TTFields is approved by the FDA for both recurrent and newly diagnosed GBM. In this study, we conducted a comprehensive review and analysis of single‐center clinical data from newly diagnosed HGG patients. Our results showed that TTFields demonstrated favorable safety and efficacy when administered to Chinese patients with HGG after completion of radio‐chemotherapy, providing greater confidence in its use in the Chinese population. To further explore the therapeutic potential of TTFields in HGG patients, future studies should aim to accumulate a larger cohort of patients.

## AUTHOR CONTRIBUTIONS

**Li Zhang:** Conceptualization (equal); data curation (equal); formal analysis (lead); methodology (equal); software (lead); visualization (lead); writing – original draft (lead); writing – review and editing (equal). **Yanming Ren:** Data curation (equal); formal analysis (equal); investigation (lead); methodology (lead); resources (lead); software (equal); validation (equal); writing – original draft (equal). **Youheng Peng:** Conceptualization (equal); data curation (equal); investigation (equal); resources (equal); visualization (equal). **Yong Luo:** Investigation (supporting); methodology (supporting); resources (equal). **Yanhui Liu:** Resources (equal); validation (equal). **Xiang Wang:** Resources (equal); validation (equal). **Yuan Yang:** Formal analysis (equal); investigation (equal); validation (equal). **Lei Liu:** Methodology (equal); validation (equal). **Ping Ai:** Investigation (equal); resources (equal). **Xiaoyan Yang:** Data curation (equal); methodology (equal). **Yanchu Li:** Methodology (equal); resources (equal). **Qing Mao:** Project administration (equal); resources (equal); supervision (equal); validation (equal). **Feng Wang:** Conceptualization (equal); project administration (lead); resources (equal); supervision (lead); writing – review and editing (lead).

## FUNDING INFORMATION

No funding was received for conducting this study.

## CONFLICT OF INTEREST STATEMENT

The authors declare that they have no conflict of interest.

## ETHICS STATEMENT

The study was approved by the Ethics Committee on Biomedical Research, West China Hospital of Sichuan University, and a waiver of informed consent was obtained.

## Supporting information


Table S1.

Table S2.

## Data Availability

The data that supports the findings of this study are available in the supplementary material of this article.
